# Congenital duodenal web causing partial obstruction with recurrent vomiting and abdominal distention in a toddler boy: a case report

**DOI:** 10.1186/s13256-023-04179-3

**Published:** 2023-11-24

**Authors:** Khesrow Ekram, Farida Razawi, Sayed Najmuddin Jalal, Mohammad Nader Sultani, Abdul Wali Wali, Mohammad Sharif Sediqi, Abdul Ghafar Hamdard, Massoud Hemat, Mohammad Mustafa Sahibzada

**Affiliations:** 1 Department of Pediatrics, Maiwand Teaching Hospital, Kabul University of Medical Sciences, 1st District, Jada-e-Maiwand, 1001 Kabul, Afghanistan; 2 Department of Pediatric Surgery, Kabul University of Medical Sciences, Kabul, Afghanistan; 3 Department of Dermatology, Kabul University of Medical Sciences, Kabul, Afghanistan

**Keywords:** Duodenal web, Duodenal obstruction, Congenital, Recurrent vomiting

## Abstract

**Background:**

The duodenal web is a thin, elongated, web-like structure that is one of the factors contributing to duodenal obstruction. Only 100 cases have been reported in the literature. We present a 2.5-year-old cachectic Afghan child who did not have any overt signs and symptoms of intestinal obstruction, like recurrent vomiting, abdominal distention, and weight loss. The web was discovered near the intersection of the third and fourth portions, which is an uncommon location for the duodenal web. The late presentation of congenital duodenal web with partial obstruction is rare but well-known and has been reported in this case.

**Case presentation:**

A 2.5-year-old cachectic Afghan child who had recurrent vomiting and experienced abdominal distention was brought to Maiwand Teaching Hospital from the Jabelsuraj region of Parwan province. The patient was suffering from unusual signs and symptoms like recurrent vomiting, abdominal distention, weight loss, and constipation. The diagnosis of these anomalies was established by a detailed history, clinical features, and abdominal CT scan. In the computerized tomography scanning (CT-Scan) image reported, there was a web with stenosis and partial obstruction in the distal aspect of the third-to-fourth portion of the duodenum. After preoperative stabilization, the child was taken for surgery. The abdomen was opened by a right upper abdominal transverse incision. After web resection and duodenoplasty, the patient was shifted to the recovery room in satisfactory condition. The child was allowed to feed after 8 days, which he tolerated well.

**Conclusion:**

Congenital duodenal web with partial obstruction is typically observed in the second and third years of life. It is suspected in patients with recurrent vomiting, abdominal distention, weight loss, and constipation. Partial obstruction may not have an overt presentation, making it a challenging diagnosis for general practitioners. Abdomen X-ray and CT scan usually confirm the diagnosis, and successful surgical intervention is recommended.

## Introduction

A duodenal web, which is one of the causes of duodenal obstruction, is a thin, round, partially permeable, web-like structure. It is a rare condition; with approximately 1 in 10,000 to 1 in 40,000 cases reported in the literature [1–3, 8].Congenital duodenal web with partial obstruction may present with intermittent symptoms that progress until obstruction is complete. Vomiting is the cardinal symptom. Initially, patients may demonstrate better tolerance to liquids than solid food. In a later stage, patients may develop significant weight loss due to poor caloric intake. When obstruction persists, patients may develop significant and progressive gastric dilatation. The stomach eventually loses its contractility. Undigested food accumulates and may represent a constant risk for aspiration pneumonia. The severity of symptoms and the age of onset vary according to the size and location of the web and if the obstruction is total or partial. Therefore, partial obstruction due to duodenal web may have a late presentation and a more challenging diagnosis [3, 7, 8, 10, 11].The second part of the duodenum is the most common site, representing 85% to 90% of all duodenal web cases. The third and fourth parts of the duodenum represent 20% and 10% of DW cases, respectively [5–7]. In addition the diagnosis of DW with partial obstruction was based on clinical presentation, abdominal X-ray and CT scan, flexible endoscopy, and an upper gastrointestinal barium study. [2, 5, 10]. Surgical treatment (either open or endoscopic approaches) is the first-line choice. However, on rare occasions, duodenal web can have a distinctly different pattern of presentation, with a delayed onset during late infancy or early childhood and various symptomologies [1, 3, 10]. Herein, we report a case of a 2.5-year-old cachectic Afghan child who presented with symptoms of recurrent vomiting, abdominal distention, and weight loss since the age of 1 year and was found to have a duodenal mucosal web on the exploratory laparotomy.

## Case report

A 2.5-year-old cachectic Afghan child who was vomiting and experiencing abdominal distention was brought to Maiwand Teaching Hospital from the Jabulseraj region of Parwan province. The patient was suffering from fever, recurrent vomiting, abdominal distention, weight loss, constipation, and painful defecation. The patient's parents were consanguineous and had a normal prenatal history. The kid experienced food intolerance at this time, and he vomited four to five times daily, forcefully and non-biliously, often 30 min to an hour after feeds, according to his mother's explanation. The patient also experienced constipation, fever, and stomach distention at the age of one year. If he was fed primarily solids, his vomiting would worsen. Due to this, at the age of 2.5 years, his diet was primarily composed of milk and small portions of semisolid food.

His parents took him frequently to their local general practitioners, who would give him medical treatment, but no remedy was achieved. As he did not have any overt signs and symptoms of intestinal obstruction, the local general practitioner did not think of the underlying organic cause of the vomiting. The parents were concerned as the vomiting was persistent and associated with weight loss. His parents added that they took the child once outside the country for better treatment at the age of 1.5 years. The reason for his referral to the capital and then to our pediatric service was the ongoing deterioration of his condition.

He is the seventh child of his parents; he was born normally at term, and since birth, he has received on-demand breast milk. At 7 months old, the mother tried complementary feeding, but she was unsuccessful due to the infant's ongoing vomiting.

In the family history, his two brothers suffered duodenal web and passed away during surgery.

On physical examination, in the abdomen, bowel sounds were normal. There was no enlargement of the liver and spleen, but the abdomen was distended. The rest of the physical findings were normal.

**Fig. 1:**
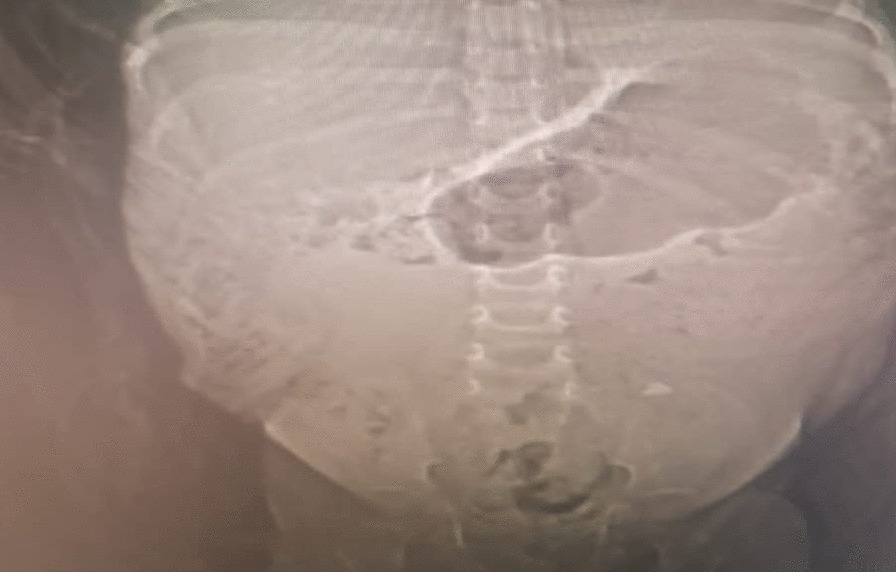
The abdominal X-ray showed marked distension in the stomach and duodenum

On the imaging study, the upright abdominal X-ray showed marked distension in the stomach and duodenum, and the ‘’double bubble’’ sign was observed. The gas detected within the distal intestinal loops pointed to a partial obstruction (Fig. 1).

**Fig. 2:**
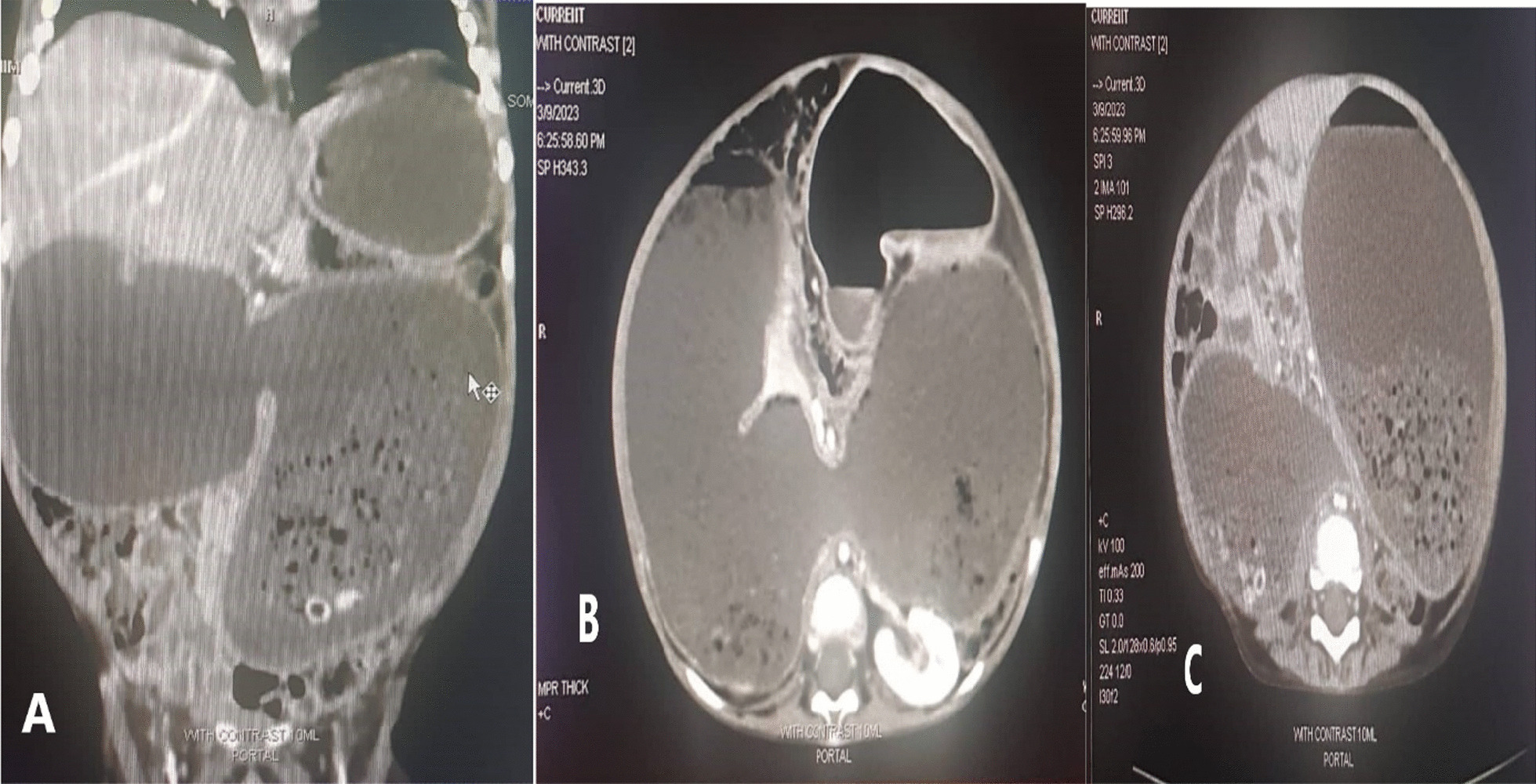
**A** coronal**,** **B** sagittal, and **C** axial A view **of** the abdominal computerized tomography showed stenosis in the distal aspect of the third and fourth segments of the duodenum

The abdominal CT scan with contrast noted severe distension in the stomach and duodenum; furthermore, there was stenosis in the distal aspect of the third-to-fourth portion of the duodenum, as well as distension in the first and second portions. The web between the third and fourth portions of the duodenum was present, but the distal intestinal loops still showed contrast, suggesting a partial obstruction there (Fig. 2).

**Fig. 3:**
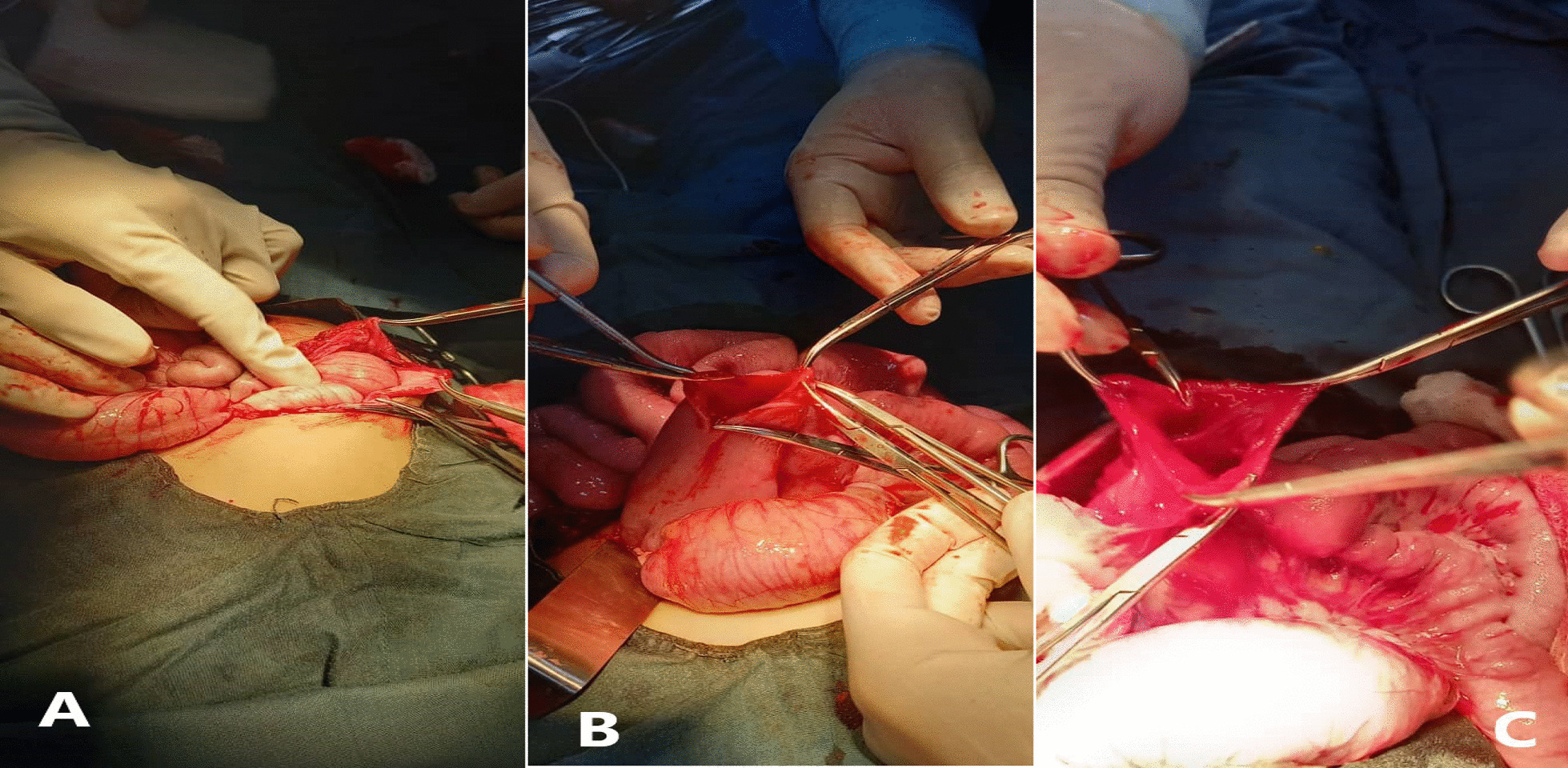
**A** abdomen is opened by a right upper transfer incision, **B** the duodenum was much dilated, and the web was located between the third and fourth parts of the duodenum, and **C** the web was identified and excision

The patient was admitted to the surgical ward, the parents were counseled, and informed consent was taken for an exploratory laparotomy. The abdomen was opened by the right upper transverse incision. Between the third and fourth portions of the duodenum, a large duodenal bulb with distal collapse was discovered. There were small food particles like beans, nuts, and seeds of the date palm in the dilated part of the duodenum. The web was removed, duodenoplasty was performed, and the abdomen was then sutured layer by layer. Intravenous fluids, broad-spectrum antibiotics (ceftriaxone and metronidazole), and proton pump inhibitors were administered for 7 days. Adequate analgesia was given. An NG tube was used to decompress the stomach. NG drainage was high for 3 days postoperatively, which gradually reduced, and the NG tube was removed on the fifth postoperative day. Oral feeding was started gradually, and the patient tolerated full oral feeds on the 8th postoperative day. On the 12th day post-op, he was discharged from the hospital in good condition. The patient was scheduled for follow-up, and after 40 days of follow-up, he was doing well, accepting a normal diet, and gaining weight of about 1 kg. He had no episodes of vomiting or abdominal pain (Fig. 3).

## Discussion and conclusion

Congenital duodenal web is classified as complete or partial obstruction. According to age, the complete congenital duodenal obstruction due to the web is more common in newborns, and the partial type is seen in infancy and childhood. Based on literature, the complete form of congenital DW is reported more often than the partial DW type. [1, 2, 7].

The signs and symptoms of DW are different based on the type of obstruction (complete or partial). Symptoms of complete DW are more significant, like vomiting, abdominal distention, weight loss, dehydration, water and electrolyte imbalance, and constipation, which are seen in the neonatal period [1, 2, 9–11]. In this case report, the child did not have any overt signs and symptoms of intestinal obstruction, and the local general practitioners did not think of the underlying organic cause of the vomiting, but they misdiagnosed the case as gastroenteritis.

The signs and symptoms of partially congenital DW increase after the intake of complementary foods [6, 8]. In this case, however, the patient could tolerate a small amount of milk and fluid and was gradually losing weight daily. The problem was increased by the occlusion of the duodenum lumen due to small food particles such as beans, nuts, and dates, which were observed during the surgery.

The majority of the duodenal web (85% to 90%) occurs in the first and second portions of the duodenum. However, the third and fourth parts of the duodenum represent 20% and 10% of WD cases, respectively. [5–7]. In this finding, the obstruction was seen in the 3rd and 4th portions of the duodenum.

50% of congenital DW is associated with other anomalies like Down syndrome, CHD, gut malrotation, hypospadias, inguinal hernia, cleft palate, and microophthalmia with pthysis bulbi [2, 6, 7], but this patient did not have these anomalies.

According to the medical literature, few cases of DW-related deaths have been reported, and most of them had other anomalies such as Down syndrome and CHD [4], but this case was not associated with any anomalies; the surgery was successfully done, the patient was discharged in good condition, and he was asked for follow-up after 40 days. After follow-up, it was noticed that the vomiting, abdominal distention, and constipation had disappeared, the patient had gained about 1 kg, and his overall condition had gotten much better.

Most cases of DW can be easily diagnosed by a simple X-ray and abdominal CT scan with contrast [2, 5, 10], but in this case, because the overt signs and symptoms were not present due to partial obstruction, the local general practitioners did not recommend diagnostic exams. When the patient was admitted to Maiwand Teaching Hospital, we recommended an abdominal CT scan with contrast. The result of the CT showed severe distension in the stomach and duodenum; besides, web was reported to be seen in the 3rd and 4th portions of the duodenum.

Congenital DW with partial obstructions in children occurred in both genders, male and female [1–5, 9]; in this case report, congenital duodenal web with partial obstruction is seen in a male child patient.

The final diagnosis of DW was made after the surgery procedure, the investigation of the area, and seeing the webs.

In the differential diagnosis of DW, other diseases such as congenital pyloric stenosis, duodenal atresia, annular pancreas, superior mesenteric artery syndrome, periduodenal portal vein, duodenal duplication cysts, and midgut volvulus must be considered. [3, 4, 7]. The detailed history, physical examination, imaging examination ‌ (CT scan, ultrasound, endoscopy, X-ray), and laboratory exams are done to ensure a final and accurate diagnosis of congenital duodenal web.

In conclusion, in this case, the patient did not have specific signs and symptoms of DW due to the partial obstruction, which occurred in a later stage of childhood, and the web being attached to the 3rd and 4th portions of the duodenum. In developing countries such as Afghanistan, due to a lack of access to equipped medical services, most such cases are put aside and not considered specifically.

Our recommendation for local general practitioners is that when they encounter such cases of recurrent vomiting, abdominal distention, constipation, and weight loss, the diagnosis of DW should be kept in mind, and the patient must be referred to a well-equipped medical facility for further investigation and treatment.

## Data Availability

The datasets used in the current article are available from the corresponding author upon reasonable request.

## Abbreviations

DW: Duodenal web
IV: Intera venous
CT: Computerized tomography
OT: Operation theater
GI: Gastrointestinal
NGT: Naso Gastric Tube
CHD: Congenital Heart Disease
